# The pathway to internship success: how academic self-concept and engagement shape outcomes in higher education

**DOI:** 10.3389/fpsyg.2026.1924391

**Published:** 2026-10-02

**Authors:** Yongrui Cui

**Affiliations:** Computer Science and Engineering College, Dalian Minzu University, Dalian, Liaoning, China

**Keywords:** academic self-concept, Chinese universities, internship engagement, internship outcomes, structural equation modeling

## Abstract

**Background:**

Internships connect academic learning with workplace practice, yet the psychological process through which students' academic self-perceptions are associated with internship success remains insufficiently specified. This study examined whether multidimensional internship engagement statistically mediates the association between academic self-concept and internship outcomes.

**Methods:**

A quantitative, multi-institutional, three-wave time-lagged survey was conducted with 522 undergraduate students from Chinese universities. Academic self-concept was assessed at internship entry, internship engagement at the internship midpoint, and internship outcomes after completion. Established instruments were adapted through forward translation, back-translation, expert review, and pilot testing. Confirmatory factor analysis, latent-variable structural equation modeling, 5,000-resample bootstrap mediation analysis, full-information maximum likelihood, robustness checks, and subgroup analyses were used.

**Results:**

The hypothesized three-factor measurement model fit better than the two-factor and one-factor alternatives. The structural model showed acceptable fit (CFI = 0.958, TLI = 0.953, RMSEA = 0.034, and SRMR = 0.044). Academic self-concept was positively associated with internship engagement, and internship engagement was positively associated with internship outcomes. The indirect association through internship engagement was 0.249 (95% bootstrap confidence interval: 0.184 to 0.319). The proportion mediated was 50.6% (95% bootstrap confidence interval: 39.4% to 62.6%), and the direct association remained statistically significant. The pattern was stable across alternative specifications and exploratory subgroup analyses.

**Conclusion:**

Internship engagement statistically accounted for part of the association between academic self-concept and internship outcomes. The findings support a theoretically ordered, but not causal, pathway in which students' academic self-evaluations are associated with stronger behavioral, cognitive, and emotional engagement during workplace learning.

## 1 Introduction

The integration of internships into undergraduate curricula has become an important component of higher education because internships connect classroom learning with workplace practice and support professional competence, career readiness, and discipline-specific application (Henseler et al., 2014; Fornell and Larcker, 1981). Internship quality is, therefore, increasingly evaluated not only by placement completion or exposure, but also by how students interpret, engage with, and apply academic preparation in professional settings (Preacher and Hayes, 2008; Hu and Bentler, 1999). Academic self-concept and internship engagement are relevant to this process. Academic self-concept concerns students' evaluative perceptions of their academic competence (Anderson and Gerbing, 1988; Chen and Gan, 2021), whereas internship engagement concerns behavioral, cognitive, and emotional participation in workplace learning (Luo et al., 2023; Picton and Baik, 2024; Fredricks et al., 2004). Placement exposure alone, however, does not explain why students in similar settings achieve different developmental outcomes (Nunley et al., 2016).

Internship research has identified several determinants of placement quality and effectiveness (Maertz et al., 2014). Prior studies have linked internships with employment opportunities, job marketability, satisfaction, skill acquisition, supervisor support, task relevance, and institutional coordination (D'Abate et al., 2017; Baert et al., 2021; Narayanan et al., 2010; Gault et al., 2010; Ferla et al., 2009). These findings establish that internship outcomes depend on more than duration or sector. They also depend on how students use the available learning opportunities. Nevertheless, many studies compare students with and without internship experience or assess satisfaction only after completion, providing limited evidence about the within-person psychological process that unfolds during the internship.

Academic self-concept must also be distinguished from academic self-efficacy. Academic self-concept is a comparatively broad, self-descriptive, and evaluative judgment about one's competence in an academic domain. Academic self-efficacy is more task- and situation-specific and concerns whether a person believes that a particular action can be executed successfully. Although the constructs are related, they differ in specificity, temporal stability, and reference frame (Marsh and Martin, 2011; Marsh and Craven, 2006; Bong and Skaalvik, 2003; Shen et al., 2024; Supervía et al., 2020). This distinction is central to the present model: a relatively broad academic self-evaluation may shape students' readiness to invest effort across varied internship tasks, whereas task-specific efficacy may fluctuate from one workplace demand to another.

Related mediation pathways have been reported in educational research. For example, learning engagement has been examined as a mediator between self-efficacy and academic achievement, and internship effectiveness has been linked with vocational identity and employability confidence (Luo et al., 2023; Zou et al., 2024). The present study therefore does not claim that engagement mediation is absent from the international literature. Its incremental contribution is narrower: it tests academic self-concept, rather than task-specific self-efficacy, as the antecedent; specifies internship engagement as a multidimensional workplace-learning construct; separates predictor, mediator, and outcome across three measurement waves; and evaluates the pathway among Chinese undergraduates.

The proposed pathway is consistent with complementary social-cognitive and control-value accounts of engagement. Students who evaluate themselves as academically capable are more likely to interpret demanding tasks as manageable, set approach-oriented goals, persist after setbacks, seek feedback, and use deeper cognitive strategies. These appraisals are expected to support behavioral engagement through effort and participation, cognitive engagement through knowledge integration and self-regulation, and emotional engagement through interest and lower withdrawal. Engagement, in turn, increases the likelihood that workplace experiences are converted into demonstrable learning and developmental outcomes (Guo et al., 2022; Ye et al., 2025).

Internship engagement is, therefore, conceptualized as a candidate statistical pathway linking academic self-concept with internship outcomes, not as the only possible explanation. Existing studies have examined related sequences involving self-efficacy, professional identity, goal orientation, adaptability, and engagement (Lin et al., 2023; Luo et al., 2023). The present model isolates one theoretically ordered pathway while recognizing that self-regulation, career adaptability, professional identity, supervisor support, and task relevance may operate in parallel or in sequence. A partial-mediation specification is consequently more plausible than a direct-only or complete-mediation model.

The pathway is particularly relevant to Chinese higher education, where applied learning, employability development, university-industry collaboration, and credit-bearing internships have received increasing attention. Chinese-context studies have reported associations among internship participation (Gutierrez et al., 2026), self-efficacy, engagement, professional identity, employability confidence, and academic outcomes (Chen and Gan, 2021; Luo et al., 2023; Ye et al., 2025; Lin et al., 2023; Alifiana et al., 2026). These studies provide an important contextual foundation, but they vary in discipline, construct definition, and design. The present study extends this evidence by examining multiple university types and regions and by temporally separating academic self-concept, internship engagement, and internship outcomes.

Previous internship studies have frequently used cross-sectional, single-institution designs or composite measures that do not clearly distinguish predictors, mediators, and outcomes (Lin et al., 2023). Such designs constrain temporal interpretation, measurement precision, and contextual coverage. The present study therefore used a multi-institutional, three-wave design, confirmatory factor analysis, latent-variable structural equation modeling, bootstrapped mediation testing, missing-data sensitivity analyses, supervisor-rating adjustment, and exploratory subgroup analyses (Bong and Skaalvik, 2003). The study evaluates theoretically ordered associations rather than causal effects and defines its contribution in terms of design, construct specificity, and Chinese-context evidence.

- It develops and tests an integrated mediation model linking academic self-concept, internship engagement, and internship outcomes in the context of Chinese undergraduate education.
- It extends academic self-concept research beyond classroom achievement by examining its relevance to workplace-based learning and internship outcomes.
- It advances internship and work-integrated learning research by evaluating internship engagement as a candidate statistical pathway through which academic self-perceptions are associated with professional and developmental outcomes.
- It applies a three-wave, multi-institutional design that strengthens temporal ordering among predictor, mediator, and outcome constructs while retaining an explicitly observational interpretation.
- It uses latent-variable structural equation modeling, confirmatory factor analysis, and bootstrapped mediation testing to improve measurement precision and statistical rigor.
- It evaluates the robustness of the mediation pathway across alternative model specifications, missing-data approaches, supervisor-rating adjustment, and subgroup analyses.
- It provides practical evidence for universities seeking to improve internship quality by strengthening both students' academic self-concept and their behavioral, cognitive, and emotional engagement during internship experiences.

## 2 Methods

### 2.1 Research design

The study used a quantitative, multi-institutional, three-wave time-lagged survey design to examine the statistical mediating role of internship engagement in the association between academic self-concept and internship outcomes among undergraduate students in China. The design was explanatory and observational. Academic self-concept was measured during the first week of the internship (Time 1), internship engagement was measured near the internship midpoint, generally during weeks 4–6 (Time 2), and internship outcomes were measured within 2 weeks after internship completion (Time 3). Structural equation modeling was used to estimate direct, indirect, and total associations among the focal constructs. The temporal separation strengthens temporal ordering and reduces same-occasion common-method variance, but it does not establish causality.

The analytical model was based on the premise that students with more positive academic self-evaluations would be more likely to participate actively in workplace tasks, apply academic knowledge, persist when difficulties emerged, seek feedback, and maintain cognitive and emotional investment. Internship engagement was therefore specified as an intervening variable between baseline academic self-concept and end-of-internship performance, professional development, career readiness, workplace adaptation, and satisfaction. Reciprocal or omitted-variable explanations remained possible and were considered when interpreting the results.

### 2.2 Research setting and participants

The target population comprised full-time undergraduates completing credit-bearing or curriculum-required internships of at least 4 weeks in education, business, engineering, information technology, health-related fields, or the social sciences. The sampling frame included 12 participating universities: four in eastern China, four in central China, and four in western China. Within each region, two comprehensive universities and two applied undergraduate institutions were included to increase institutional diversity. University participation depended on institutional access and willingness; consequently, the design is most accurately described as stratified, multi-institutional recruitment with a convenience component at the institutional level.

Recruitment quotas were set by region, university type, academic discipline, and year of study. Internship offices generated lists of eligible students within each stratum. Where complete rosters were available, students were selected using computer-generated random numbers; where rosters could not be released, all eligible students in selected internship cohorts were invited consecutively. The achieved regional and institutional distributions were monitored against the pre-specified quotas, and deviations did not exceed 4.1 percentage points. Inclusion criteria were full-time undergraduate status, enrollment in a credit-bearing or curriculum-related internship, a planned internship duration of at least four weeks, completion of the Time 1 survey during the first internship week, and capacity to provide informed consent. Students were excluded if the placement was unrelated to their program, the internship was non-credit-bearing, the three-wave linkage code could not be verified, or responses showed duplication, failed attention checks, excessive missingness, or invalid patterns.

Sample-size planning used a Monte Carlo mediation simulation with anticipated standardized constituent paths of 0.30, a residual direct association of 0.20, a two-sided significance level of 0.05, and 5,000 simulated replications. A minimum of 458 complete cases was estimated to provide at least 80% power for the indirect association while maintaining stable estimation of the 23-indicator, three-factor model. The target was increased to approximately 500 valid cases to accommodate model complexity, subgroup analyses, and incomplete supervisor ratings. Seven hundred students were invited at baseline to allow for nonresponse and attrition across the three survey waves; the final analytical sample of 522 exceeded the planned minimum.

### 2.3 Conceptual model and hypotheses

The conceptual model specified academic self-concept as the independent latent variable, internship engagement as the mediating latent variable, and internship outcomes as the dependent latent variable. Internship engagement comprised behavioral, cognitive, and emotional dimensions. The primary internship-outcome factor comprised self-reported performance, professional skill development, career readiness, workplace adaptation, and satisfaction. A separate five-item supervisor rating was available for a subset of participants and was used only in a robustness specification rather than as an indicator of the primary latent outcome.

Four hypotheses were tested. H1: Academic self-concept at Time 1 will be positively associated with internship outcomes at Time 3. H2: Academic self-concept at Time 1 will positively predict internship engagement at Time 2. H3: internship engagement at Time 2 will positively predict internship outcomes at Time 3. H4: internship engagement will carry a statistically significant indirect association between academic self-concept and internship outcomes, while a residual direct association may remain. The main constructs, operational definitions, dimensions, and analytical roles are summarized in Table 1.

**Table 1:** Main constructs, operational definitions, and analytical roles.

Construct	Operational definition	Main dimensions	Analytical role
Academic self-concept	Students' perception of their academic competence, learning ability, and confidence in handling university-level academic tasks	Academic competence; learning confidence; perceived academic capability	Independent variable
Internship engagement	Students' behavioral, cognitive, and emotional involvement in internship tasks and workplace learning	Behavioral engagement; cognitive engagement; emotional engagement	Mediating variable
Internship outcomes	Students' internship-related performance and developmental gains after internship completion	Internship performance; professional skill development; career readiness; internship satisfaction	Dependent variable
Control variables	Individual, academic, and internship-related characteristics that may affect engagement or outcomes	Gender; age; academic year; major; university type; prior GPA; internship duration; internship sector	Covariates

### 2.4 Measures and data collection

The measures were adapted from established instruments rather than developed de novo. All student-reported items used a common five-point response scale ranging from 1 (strongly disagree) to 5 (strongly agree). The source instruments used different response formats; a common five-point format was selected to reduce response burden across three waves, simplify bilingual administration, and maintain a consistent metric across constructs. Two bilingual researchers independently completed forward translations, a third bilingual researcher completed the back-translation, and discrepancies were resolved by consensus. A five-member panel with expertise in educational psychology, work-integrated learning, and psychometrics evaluated content equivalence and clarity. Item-level content-validity indices ranged from 0.80 to 1.00, and the scale-level average content-validity index was 0.94. The translated instrument was piloted with 38 students who were not included in the analytical sample. The exact item wording, construct dimension, administration wave, and source mapping are provided in Supplementary Table S1.

Academic self-concept was measured at Time 1 using six items adapted from academic self-concept and self-belief measures (Marsh and Craven, 2006; Bong and Skaalvik, 2003; Shen et al., 2024; Supervía et al., 2020). The items assessed perceived academic competence, confidence in mastering discipline-specific content, comparative academic capability, and the perceived ability to apply academic knowledge. Higher scores indicated a more positive academic self-concept. Internship engagement was measured at Time 2 using nine items: three behavioral, three cognitive, and three emotional engagement indicators. Item development and adaptation were guided by multidimensional engagement frameworks and related Chinese-college evidence (Luo et al., 2023; Picton and Baik, 2024; Fredricks et al., 2004; Guo et al., 2022; Lin et al., 2023). Higher scores indicated greater effort and participation, deeper cognitive investment, and stronger positive emotional connection to workplace learning. Internship outcomes were measured at Time 3 using eight student-reported items adapted from internship effectiveness, satisfaction, career-readiness, and developmental-outcome measures (Baert et al., 2021; Narayanan et al., 2010; Ferla et al., 2009). The indicators covered task performance, professional skill development, career readiness, workplace adaptation, and overall satisfaction. Supervisors independently completed five parallel performance items for 407 students (78.0%); these ratings were linked using anonymous identification codes and were used in a robustness model.

Control variables were selected because prior achievement, demographic characteristics, institutional setting, and placement structure may be associated with both engagement and outcomes. Gender, academic year, major, university type, region, internship sector, and payment status were dummy coded. Age, prior grade point average, and internship duration were entered as grand-mean-centered observed covariates. All controls were specified as predictors of both internship engagement and internship outcomes; the unadjusted model was retained as a robustness check. Prior internship experience, motivation, personality, supervisor support, and task relevance were not measured and are acknowledged as possible omitted variables.

Composite scores were calculated by summing valid item responses and dividing by the number of valid items, as defined in Equation 1. Data from the three waves were linked using anonymous codes. Responses were screened for duplicates, failed attention checks, long-string responding, implausibly short completion time, excessive missingness, and multivariate outliers. Full-information maximum likelihood was the primary missing-data method because it retains all available observations under a missing-at-random assumption. Complete-case estimation and multiple imputation with 20 datasets were used as sensitivity analyses.


$$\begin{array}{l} S_{i}=\frac{1}{J}\overset{J}{\underset{j=1}{\sum }}x_{ij} \end{array}$$ (1)

#### 2.4.1 Measurement quality

Internal consistency was evaluated using Cronbach's alpha and composite reliability, with values of at least 0.70 considered acceptable. Convergent validity was assessed using standardized factor loadings and average variance extracted, with loadings of at least 0.50 and average variance extracted of at least 0.50. Discriminant validity was examined using the Fornell-Larcker criterion and heterotrait–monotrait ratios below 0.85. Confirmatory factor analysis was used to compare one-factor, two-factor, and hypothesized three-factor measurement structures, as expressed in Equation 2.


$$\begin{array}{l} x_{i}=\Lambda \eta_{i}+\epsilon_{i} \end{array}$$ (2)

Where *x*_*i*_ is the vector of observed indicators, Λ is the factor-loading matrix, η_*i*_ is the vector of latent constructs, and ϵ_*i*_ is measurement error.

#### 2.4.2 Mediation model

Structural equation modeling was used to estimate the mediation model. Let *X*_*i*_ denote academic self-concept, *M*_*i*_ internship engagement, *Y*_*i*_ internship outcomes, and *C*_*i*_ the vector of control variables. The mediation model is specified in Equations 3 and 4, and the indirect effect, total effect, and mediated proportion are defined in Equations 5–7, respectively.


$$\begin{array}{lll} M_{i} & = & \beta_{0}+aX_{i}+\gamma^{T}C_{i}+\epsilon_{M,i} \end{array}$$ (3)

$$\begin{array}{lll} Y_{i} & = & \beta_{1}+c^{\prime }X_{i}+bM_{i}+\delta^{T}C_{i}+\epsilon_{Y,i} \end{array}$$ (4)

Where *a* is the effect of academic self-concept on internship engagement, *b* is the effect of internship engagement on internship outcomes, and *c*′ is the direct effect after controlling for the mediator. The indirect effect, total effect, and mediated proportion were calculated as:


$$\begin{array}{lll} IE_{ind} & = & a\times b \end{array}$$ (5)

$$\begin{array}{lll} TE & = & c^{\prime }+\left(a\times b\right) \end{array}$$ (6)

$$\begin{array}{lll} PM & = & \frac{a\times b}{c^{\prime }+\left(a\times b\right)} \end{array}$$ (7)

### 2.5 Statistical analysis

The analysis proceeded in seven stages. First, descriptive statistics, distributions, correlations, and missing-data patterns were examined. Second, reliability, convergent validity, and discriminant validity were evaluated. Third, one-factor, two-factor, and three-factor confirmatory models were compared using chi-square divided by degrees of freedom, CFI, TLI, RMSEA, SRMR, AIC, BIC, and changes in CFI. Fourth, the hypothesized structural model was estimated using full-information maximum likelihood. Fifth, 5,000 bias-corrected bootstrap resamples were used to estimate confidence intervals for the indirect association, total association, and proportion mediated. Sixth, seven pre-specified robustness models were evaluated. Seventh, exploratory subgroup analyses were conducted by gender, university type, internship duration, and discipline. Statistical tests were two-sided with a significance threshold of 0.05. Standardized coefficients are reported to facilitate interpretation.

### 2.6 Ethical considerations

The study was conducted in accordance with the Declaration of Helsinki and institutional research guidelines. The institutional research committee determined that formal ethical approval was not required for this minimal-risk anonymous survey. Electronic informed consent was obtained from all participants.

Before participation, all individuals received information regarding the study purpose, procedures, time commitment, voluntary nature of participation, confidentiality measures, and their right to withdraw at any time without consequence. Electronic informed consent was obtained by selecting the “I agree to participate” option before accessing the questionnaire.

All responses were collected anonymously, and no names, student identification numbers, contact details, or other personally identifiable information were recorded. Anonymous identification codes were used solely to match student responses with supervisor evaluations while preserving participant confidentiality.

## 3 Results

### 3.1 Sample characteristics and data screening

Figure 1 shows the participant flow and data-screening process. Of the 700 students invited at baseline, 642 provided informed consent (91.7%). After sequential completion of the three survey waves, 548 students remained at Time 3 (78.3% of those invited). Quality screening excluded 26 responses because of duplicate entries, failed attention checks, excessive missingness, or invalid response patterns. The final analytical sample included 522 valid cases (74.6% of the invitation pool). Figure 1 is embedded as a high-resolution image and presents each exclusion stage from invitation to analysis.

**Figure 1:**
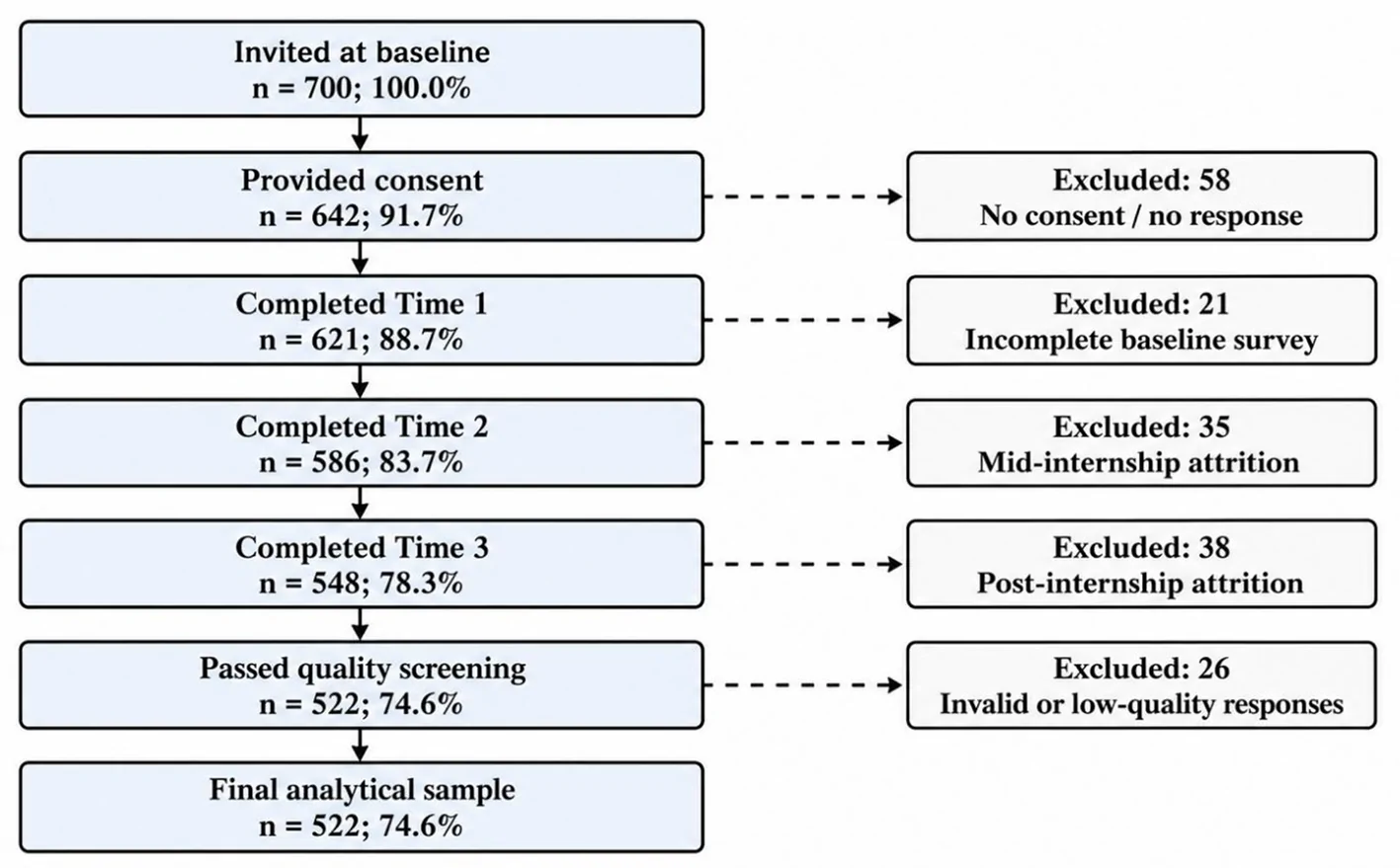
Participant flow and data-screening process.

Across retained cases, 1.6% of item-level values were missing, with item-specific proportions ranging from 0.4% to 3.1%. Little's test did not reject the assumption that the retained item-level data were missing completely at random (chi-square = 401.76, degrees of freedom = 384, and *p* = 0.255). Full-information maximum likelihood therefore served as the primary approach. The complete-case sensitivity analysis included 489 students, and the multiple-imputation analysis used 20 imputed datasets.

As shown in Table 2, the final cohort included 291 female students and 231 male students. The mean age was 21.34 years, with a standard deviation of 1.18. Most participants were in the third year or fourth year of study, accounting for 47.5% and 38.7% of the sample, respectively. Students were recruited from eastern, central, and western China, with the largest proportion coming from eastern China. Table 2 also shows that 279 students were enrolled in comprehensive universities and 243 were from applied undergraduate institutions. Academic disciplines were distributed across business and management, engineering, information technology, education, health-related majors, and social sciences. The mean prior GPA was 3.28 with a standard deviation of 0.39. The mean internship duration was 13.21 weeks, indicating that the analytical sample had completed internships of sufficient length for assessing engagement and outcomes. Regarding internship characteristics, private enterprises represented the largest internship sector, followed by public institutions, educational organizations, and healthcare or social-service organizations, as reported in Table 2. Paid internships accounted for 54.8% of the sample, whereas 45.2% were unpaid. Supervisor-rated performance data were available for 407 students, corresponding to 78.0% of the final analytical cohort.

**Table 2:** Sample characteristics of the final analytical cohort.

Variable	Category/statistic	*n*	%/Mean ±*SD*
Total sample	Valid analytical cases	522	100.0
Gender	Female	291	55.7
	Male	231	44.3
Age	Years	522	21.34 ± 1.18
Academic year	Second year	72	13.8
	Third year	248	47.5
	Fourth year	202	38.7
University region	Eastern China	201	38.5
	Central China	171	32.8
	Western China	150	28.7
University type	Comprehensive university	279	53.4
	Applied undergraduate institution	243	46.6
Academic discipline	Business and management	116	22.2
	Engineering	104	19.9
	Information technology	82	15.7
	Education	76	14.6
	Health-related majors	68	13.0
	Social sciences	76	14.6
Prior GPA	Grade point average	522	3.28 ± 0.39
Internship duration	Weeks	522	13.21 ± 4.58
Internship sector	Private enterprise	238	45.6
	Public institution	132	25.3
	Educational organization	87	16.7
	Healthcare or social-service organization	65	12.5
Internship payment status	Paid internship	286	54.8
	Unpaid internship	236	45.2
Supervisor-rated performance available	Yes	407	78.0
	No	115	22.0

The achieved distributions by region and university type remained close to the pre-specified recruitment quotas, although the eastern region was modestly overrepresented. Because universities entered through institutional cooperation and some sites used consecutive cohort invitation rather than student-level random selection, the sample should not be interpreted as nationally representative.

### 3.2 Descriptive statistics, reliability, and construct validity

Figure 2 presents the descriptive statistics and bivariate correlations for the three main study variables. Academic self-concept, internship engagement, and internship outcomes showed mean values above the midpoint of the response scale, with mean scores of 3.71, 3.83, and 3.78, respectively. The standard deviations ranged from 0.61 to 0.66, indicating moderate variability across participants. Skewness and kurtosis values were within acceptable distributional ranges for all three constructs, supporting the suitability of the data for subsequent latent-variable analyses. The correlation matrix in Figure 2 shows positive and statistically significant associations among all main constructs. Academic self-concept was positively correlated with internship engagement and internship outcomes, with correlation coefficients of 0.558 and 0.514, respectively. Internship engagement showed the strongest association with internship outcomes, with a correlation coefficient of 0.626. These results indicate that higher academic self-concept and stronger internship engagement were both associated with better internship outcomes.

**Figure 2:**
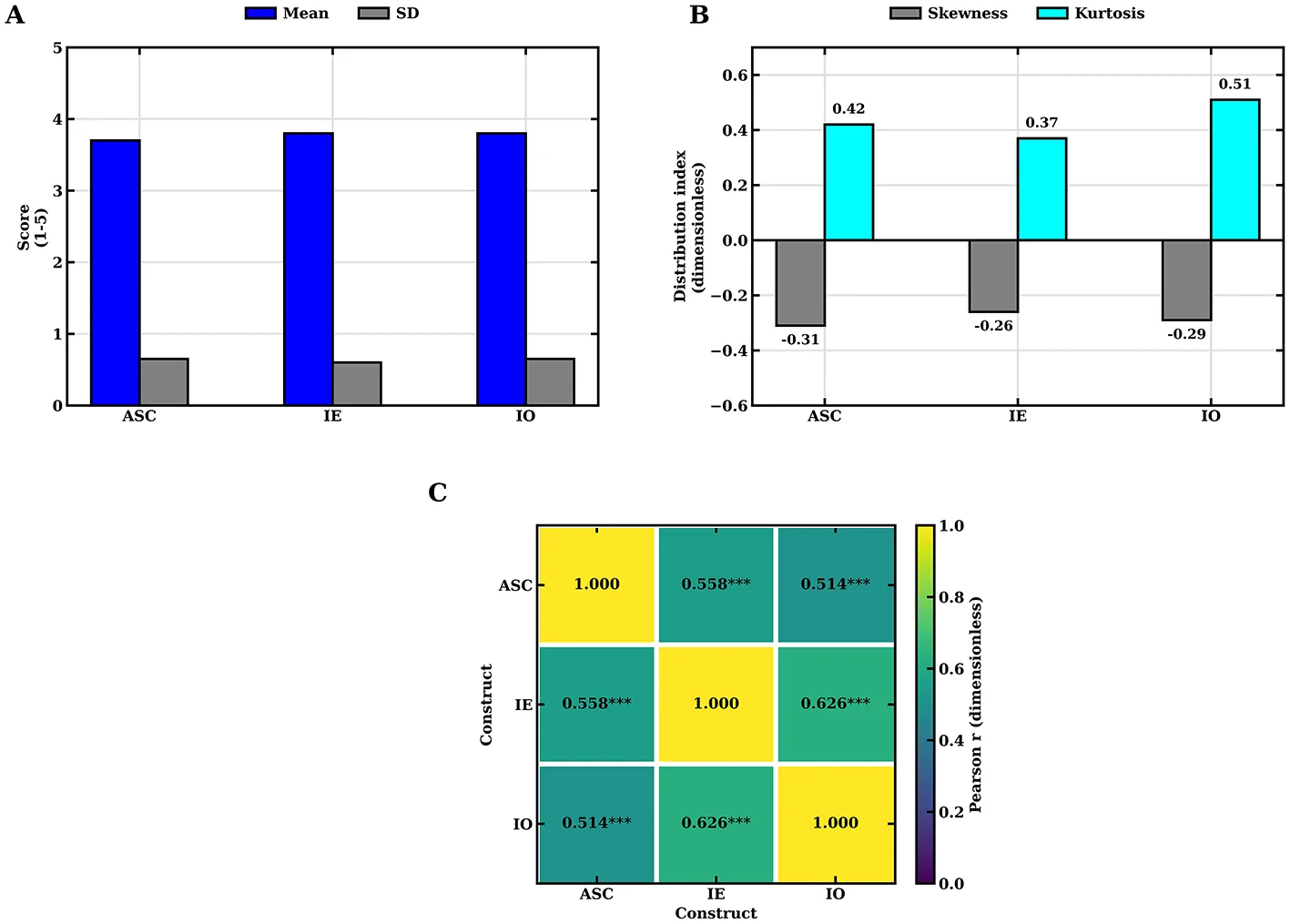
Descriptive statistics and correlation matrix. **(A)** Mean and standard deviation. **(B)** Skewness and kurtosis. **(C)** Correlations among the main constructs.

Table 3 summarizes the reliability and validity evidence for the measurement constructs. Internal consistency was satisfactory for all constructs, with Cronbach's alpha values ranging from 0.884 to 0.923 and composite reliability values ranging from 0.907 to 0.937. The standardized loading ranges and mean loading values indicated acceptable item representation for academic self-concept, internship engagement, and internship outcomes. Convergent validity was also supported, as all average variance extracted values exceeded the recommended threshold of 0.50. The square roots of AVE were higher than the corresponding inter-construct associations, and all HTMT values remained below conservative cut-off levels. As shown in Table 3, the measurement constructs demonstrated acceptable reliability, convergent validity, and discriminant validity.

**Table 3:** Reliability, convergent validity, and discriminant validity of the main constructs.

Construct	Items, *n*	Loading range	Mean loading	Cronbach's α	CR	AVE	√AVE	HTMT with ASC	HTMT with IE	HTMT with IO	Evaluation
Academic self-concept	6	0.714–0.846	0.786	0.884	0.907	0.619	0.787	–	0.624	0.579	Acceptable
Internship engagement	9	0.692–0.833	0.768	0.914	0.929	0.593	0.770	0.624	–	0.704	
Internship outcomes	8	0.721–0.862	0.793	0.923	0.937	0.628	0.793	0.579	0.704	–	

### 3.3 Confirmatory factor analysis and measurement model fit

Figure 3 presents the confirmatory factor analysis model-comparison results. The one-factor model showed poor fit, with chi-square divided by degrees of freedom = 4.46, CFI = 0.794, TLI = 0.773, RMSEA = 0.082, and SRMR = 0.093. The two-factor model improved across the major indices, with chi-square divided by degrees of freedom = 2.72, CFI = 0.898, TLI = 0.886, RMSEA = 0.058, and SRMR = 0.066, but the incremental fit indices remained below the preferred threshold. The hypothesized three-factor model showed the strongest fit, with chi-square divided by degrees of freedom = 1.66, CFI = 0.961, TLI = 0.956, RMSEA = 0.036, SRMR = 0.041, and a 90% RMSEA confidence interval of 0.030 to 0.042. Relative to the two-factor model, the three-factor model improved CFI by 0.063; relative to the one-factor model, it improved CFI by 0.167. Both changes exceeded the 0.010 criterion for meaningful improvement. The three-factor model also had the lowest information criteria (AIC = 18,428.06; BIC = 18909.11) and was retained for structural modeling.

**Figure 3:**
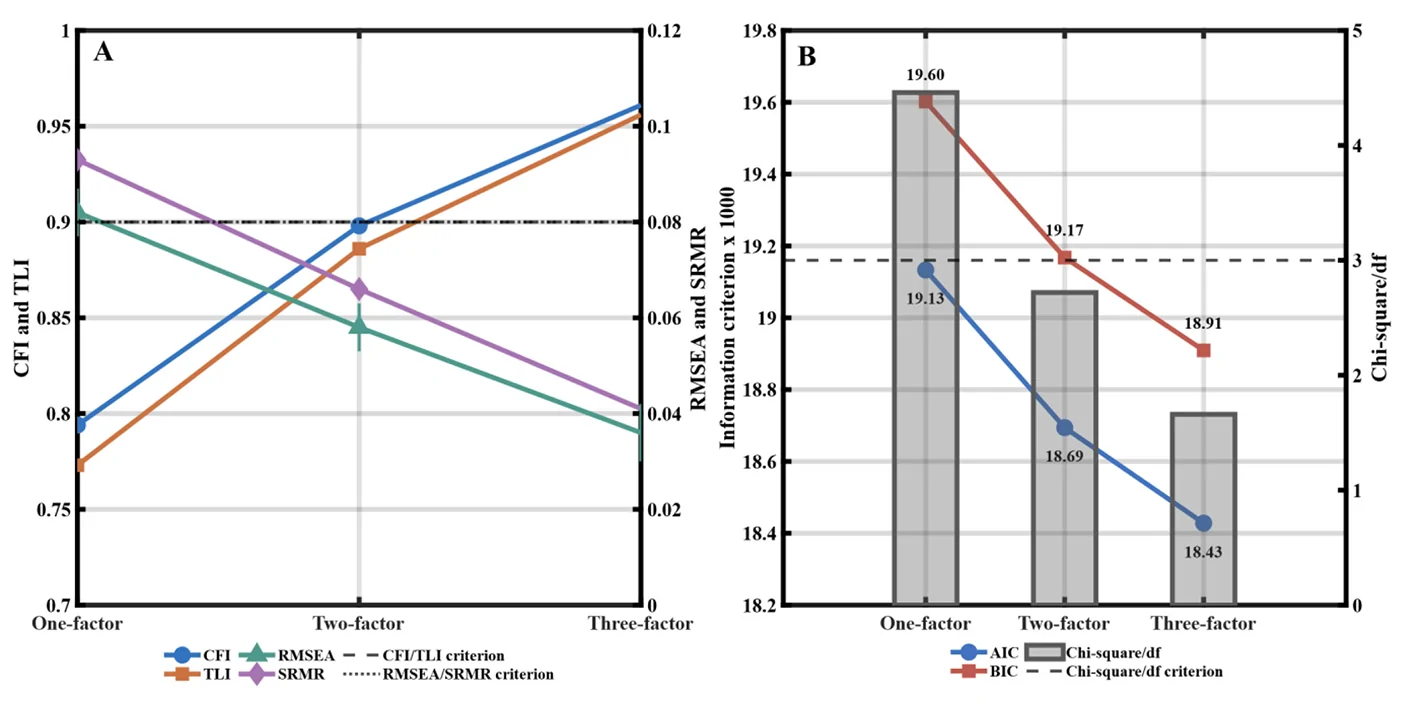
Confirmatory factor analysis and measurement model fit comparison. **(A)** Main fit indices. **(B)** Information criteria and χ^2^/df.

### 3.4 Structural model and mediation associations

Table 4 shows that the structural model achieved acceptable overall fit. The model yielded χ^2^ = 486.72 with 304 degrees of freedom, and the χ^2^/df value was 1.60. Incremental fit indices were above the recommended threshold, with CFI = 0.958 and TLI = 0.953. Residual-based indices were also within acceptable limits, with RMSEA = 0.034 and SRMR = 0.044. The 90% confidence interval for RMSEA ranged from 0.029 to 0.040. The explained variance estimates in Table 4 indicate that the model accounted for 35.1% of the variance in internship engagement and 52.4% of the variance in internship outcomes. These values show that the structural model provided adequate explanatory coverage for both endogenous constructs.

**Table 4:** Structural model fit and explained variance.

Indicator	Value	Recommended criterion	Evaluation
χ^2^	486.72	Lower values preferred	Acceptable
df	304	–	
χ^2^/df	1.60	<3.00	Acceptable
CFI	0.958	≥0.900	
TLI	0.953	≥0.900	
RMSEA	0.034	≤ 0.080	
RMSEA 90% CI	0.029–0.040	Upper bound ≤ 0.080	
SRMR	0.044	≤ 0.080	
*R*^2^ for internship engagement	0.351	Higher values preferred	Moderate explanatory power
*R*^2^ for internship outcomes	0.524	Higher values preferred	Substantial explanatory power

Figure 4 presents the standardized structural path coefficients and bootstrapped mediation estimates. Academic self-concept was positively associated with internship engagement (standardized coefficient = 0.512), and internship engagement was positively associated with internship outcomes (standardized coefficient = 0.486). The residual direct association between academic self-concept and internship outcomes remained statistically significant (standardized coefficient = 0.243). The bootstrapped indirect association through internship engagement was 0.249 (95% bootstrap confidence interval: 0.184 to 0.319), and the total association was 0.492. The ratio of the indirect to total association, equivalent to the proportion mediated, was 0.506 (95% bootstrap confidence interval: 0.394 to 0.626). Because the direct and indirect associations were both statistically significant, the pattern was consistent with partial statistical mediation.

**Figure 4:**
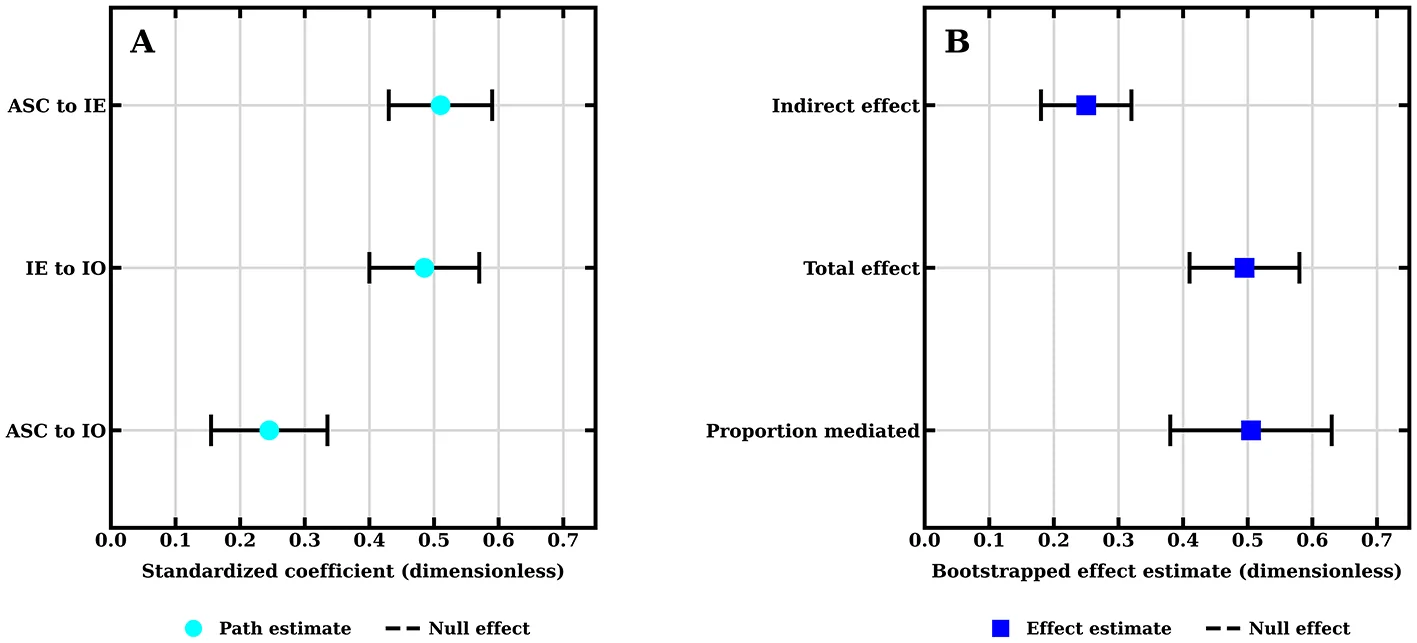
Structural path coefficients and bootstrapped mediation associations. **(A)** Structural path coefficients. **(B)** Indirect, total, and mediated associations.

### 3.5 Robustness checks and subgroup analyses

Figure 5 presents seven robustness specifications: the primary adjusted latent-variable model (M1), the latent-variable model without controls (M2), an unadjusted composite-score mediation model (M3), an adjusted composite-score model (M4), the supervisor-rating-adjusted outcome model (M5), the complete-case model (M6), and the multiple-imputation model (M7). The indirect association ranged from 0.233 to 0.295, and every bootstrap confidence interval excluded zero. The primary model yielded 0.249 (95% bootstrap confidence interval: 0.184 to 0.319), while the complete-case and multiple-imputation estimates were both 0.249. Total associations remained positive (0.470 to 0.514), proportions mediated ranged from 48.4% to 57.4%, variance explained in internship engagement ranged from 0.293 to 0.357, and variance explained in internship outcomes ranged from 0.448 to 0.526. These analyses indicate that the statistical mediation pattern was not dependent on a single scoring, covariate, supervisor-rating, or missing-data specification.

**Figure 5:**
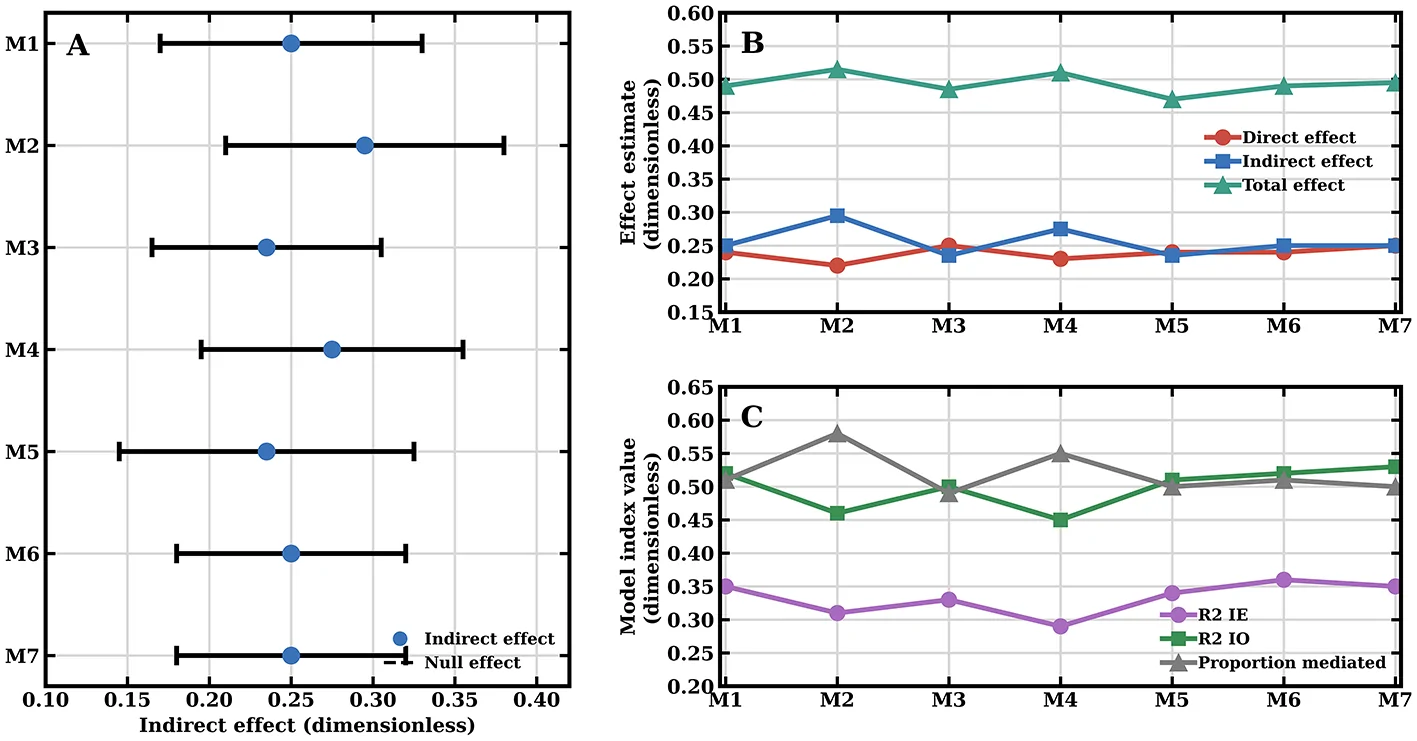
Robustness checks across alternative model specifications. **(A)** Indirect associations with 95% bootstrap confidence intervals. **(B)** Direct, indirect, and total associations. **(C)** Variance explained in internship engagement and internship outcomes and the proportion mediated.

Figure 6 presents exploratory subgroup mediation results by gender, university type, internship duration, and academic discipline. The indirect association was positive in each subgroup, and the bootstrap confidence intervals excluded zero. Estimates were 0.258 for female students and 0.237 for male students; 0.235 for comprehensive universities and 0.266 for applied undergraduate institutions; 0.217 for internships shorter than 12 weeks and 0.274 for internships of 12 weeks or longer; and 0.223 to 0.267 across discipline groups. The proportion mediated ranged from 45.1% to 55.6%. These results demonstrate consistency within the sampled subgroups but should not be interpreted as proof of equivalence or national generalizability because formal measurement-invariance and between-group difference tests were not the primary objective and some subgroup sample sizes were modest.

**Figure 6:**
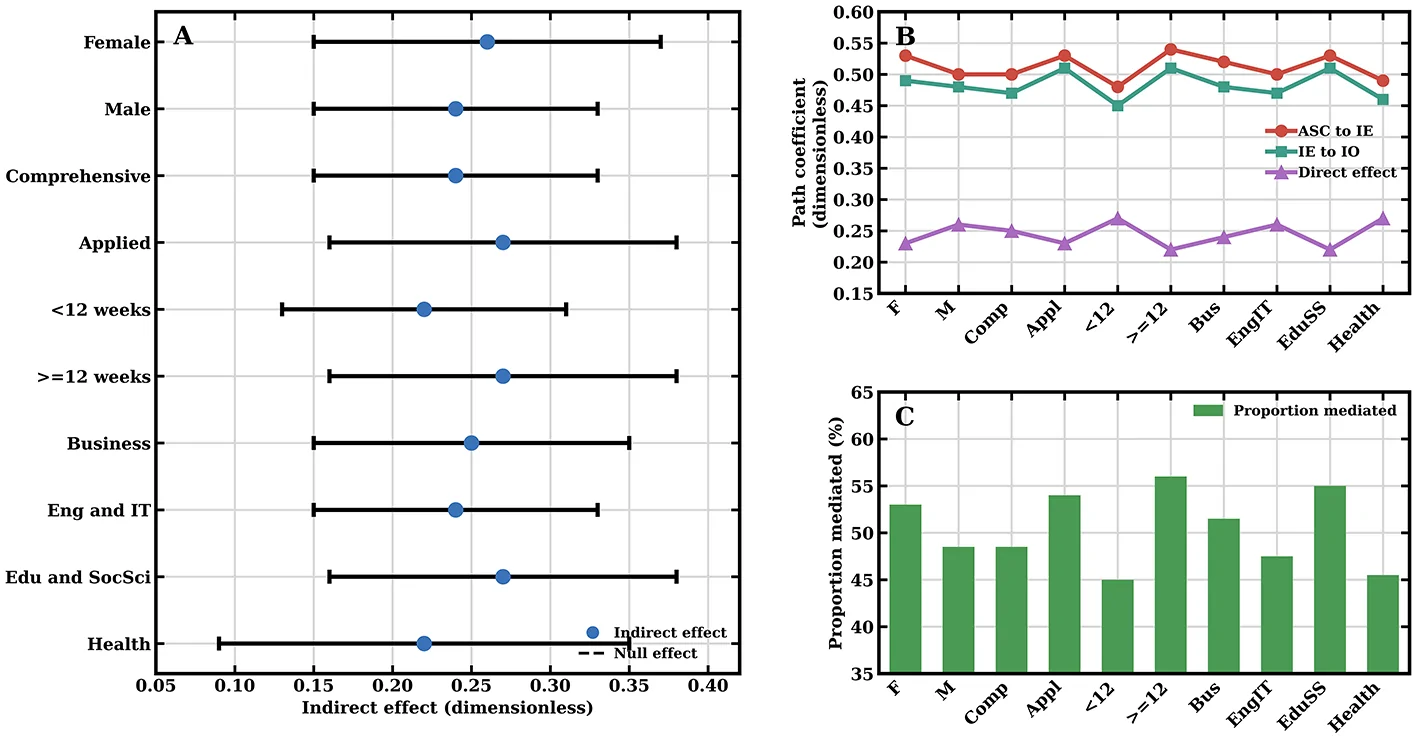
Subgroup mediation effects across participant and internship characteristics. **(A)** Indirect effects with confidence intervals. **(B)** Structural path coefficients. **(C)** Proportion mediated (%).

## 4 Discussion

The present study examined whether internship engagement statistically mediated the association between academic self-concept and internship outcomes among undergraduates in Chinese universities. Four principal findings emerged. First, the hypothesized three-factor measurement structure fit better than the one-factor and two-factor alternatives. Second, baseline academic self-concept was positively associated with mid-internship engagement. Third, internship engagement was positively associated with end-of-internship outcomes, while academic self-concept retained a positive residual association with those outcomes. Fourth, the indirect association through internship engagement was statistically significant and remained stable across alternative specifications and exploratory subgroups. Because the design was observational, these findings describe theoretically ordered associations rather than causal effects.

The positive association between academic self-concept and internship engagement is consistent with accounts that define academic self-concept as students' broad evaluations of competence, learning ability, and academic effectiveness (Ye et al., 2025). Students who regard themselves as academically capable may be more willing to accept challenging assignments, ask questions, persist after mistakes, and apply disciplinary knowledge to unfamiliar workplace problems. The distinction from self-efficacy remains important: the present predictor reflects a comparatively broad academic self-evaluation rather than confidence in executing a single internship task. The observed pathway suggests that this broader self-evaluation is relevant beyond the classroom, while not excluding a role for task-specific efficacy (Lin et al., 2023).

The positive association between internship engagement and internship outcomes further emphasizes engagement as a central component of experiential learning. In this study, internship engagement referred consistently to behavioral participation, cognitive investment, and emotional connection. Students' who invested effort, reflected on workplace problems, integrated academic knowledge, and maintained interest reported stronger performance, professional development, career readiness, adaptation, and satisfaction. This interpretation is consistent with engagement theory, which proposes that access to a learning opportunity is insufficient unless students actively use it (Gutierrez et al., 2026).

The mediation result identifies internship engagement as one plausible intervening pathway. Academic self-concept showed both a residual direct association with internship outcomes and an indirect association through engagement. This pattern is consistent with partial statistical mediation, but it does not demonstrate that engagement is the sole or causal mechanism. Self-regulation, achievement motivation, career adaptability, professional identity, prior workplace experience, supervisor support, task relevance, and organizational climate may confound, complement, or sequentially extend the observed pathway. Future studies should compare multiple-mediator models and collect repeated measures of these alternative mechanisms.

The estimated proportion mediated was 50.6%, with a 95% bootstrap confidence interval from 39.4% to 62.6%. The interval indicates substantial uncertainty around the point estimate and supports interpreting the ratio as an approximate index rather than a fixed population quantity. Nevertheless, the result suggests that internship engagement accounted statistically for a meaningful share of the total association between academic self-concept and internship outcomes. This finding helps connect academic self-concept research with work-integrated learning research, which has often emphasized workplace learning and employability outcomes separately (Alifiana et al., 2026).

The measurement findings support the distinctiveness of the three focal constructs. Reliability, composite reliability, average variance extracted, Fornell–Larcker comparisons, and heterotrait–monotrait ratios met the pre-specified criteria. The three-factor model improved CFI by 0.063 relative to the two-factor model and by 0.167 relative to the one-factor model, exceeding the 0.010 comparison criterion. Academic self-concept, internship engagement, and internship outcomes therefore represented related but empirically separable constructs rather than a single general student-success factor (Shen et al., 2024; Supervía et al., 2020).

The robustness checks provided additional support for the stability of the statistical pattern. The indirect association remained positive in latent-variable and composite-score models, adjusted and unadjusted specifications, the supervisor-rating-adjusted model, complete-case estimation, and multiple imputation. This consistency reduces, but does not eliminate, concern that the finding resulted solely from one scoring method, missing-data procedure, or outcome specification. Unmeasured confounding and reciprocal processes remain possible.

The subgroup analyses showed a similar positive pattern across gender, university type, internship-duration categories, and broad academic disciplines within the sampled institutions. These analyses suggest contextual consistency, but they do not establish universal generalizability. The sample included only Chinese undergraduates completing credit-bearing or curriculum-related internships, and the participating universities were not a probability sample of the national system. Replication is required in other countries, labor markets, institutional types, non-credit internships, postgraduate programs, and disciplines with different placement structures.

The study has three theoretical implications. First, it extends academic self-concept research by showing that broad academic self-evaluations are associated with workplace-based learning outcomes as well as classroom outcomes. Second, it specifies behavioral, cognitive, and emotional internship engagement as a statistically testable pathway between self-perception and applied learning. Third, it contributes to work-integrated learning research by integrating student psychological preparedness with the quality of participation during the placement. These implications remain provisional because the design cannot establish causal ordering or exclude competing models.

The findings support several concrete practices for universities and internship coordinators. Before placement, a 60–90-mins case-based workshop could require students to map discipline-specific knowledge onto realistic workplace scenarios, identify prior mastery experiences, and formulate two evidence-based statements about their academic strengths. At internship entry, the student, supervisor, and university coordinator could agree on three observable learning goals and define the tasks through which each goal will be practiced. During the internship, students could submit a brief weekly reflective journal linking one workplace event to academic knowledge, followed by concise supervisor feedback. Coordinators could also schedule structured check-ins at the end of weeks 2 and 6 to review task relevance, feedback access, workload, and progress toward goals.

Internship engagement can also be monitored as an intermediate program indicator. A brief engagement screen covering behavioral, cognitive, and emotional engagement could be administered during weeks 4–6. As an operational example requiring local validation, a mean score below 2.5 on the five-point scale, a decline of at least 0.5 points from an earlier check, or any dimension below 2.0 could trigger follow-up. The recommended response is a tripartite student-supervisor-university meeting within five working days, followed by a written adjustment plan addressing task clarity, feedback frequency, mentoring, workload, or opportunities to apply academic knowledge. Reassessment two weeks later would determine whether additional support or placement modification is needed.

Several limitations delimit the findings. First, participation and institutional cooperation may have introduced selection bias; participating students may have been more motivated or may have experienced more supportive placements than nonparticipants. Second, most focal measures were self-reported and may be affected by social-desirability bias, particularly when students knew that supervisors were also involved, although supervisor ratings were linked anonymously and temporal separation reduced same-occasion bias. Third, the academic self-concept measure was broad and may not fully capture domain-specific self-concept for applied workplace tasks. Fourth, supervisor ratings were unavailable for 22.0% of the sample. Fifth, time-varying factors such as supervisor support, task relevance, workload, workplace climate, motivation, and professional identity were not measured and may offer alternative explanations. Sixth, the three-wave ordering does not rule out reciprocal causation; engagement may also strengthen subsequent academic self-concept. Seventh, the sample excluded non-credit-bearing internships and was restricted to Chinese undergraduates, where institutional structures and cultural orientations toward authority, effort, and engagement may differ from other contexts. Eighth, some discipline-specific subgroups were modest, and the subgroup analyses were exploratory. Finally, the proportion mediated is a ratio statistic that can be sensitive to model specification and sampling variation, despite the bootstrap interval and robustness analyses.

Future research should use more frequent longitudinal measurement to test reciprocal change in academic self-concept and engagement throughout the internship. Experimental or quasi-experimental evaluations could examine whether case-based preparation, structured goal setting, reflective feedback, or targeted engagement support improves outcomes. Multiple-mediator models should assess self-regulation, motivation, career adaptability, professional identity, supervisor support, task relevance, and workplace climate simultaneously. Competing sequences, including engagement leading to enhanced self-concept, should be tested. Cross-national replication, measurement-invariance analysis, discipline-specific models, and studies of non-credit and postgraduate internships are also needed to establish the boundaries of the observed pattern.

## 5 Conclusion

Within this multi-institutional sample of Chinese undergraduates, higher academic self-concept at internship entry was associated with stronger internship engagement at the midpoint and more favorable outcomes after completion. Internship engagement carried a significant indirect association and accounted for an estimated 50.6% of the total association, while a residual direct association remained. The pattern was stable across alternative specifications and exploratory subgroups. Because the evidence is observational and context-specific, internship engagement should be interpreted as a plausible statistical pathway rather than a confirmed causal mechanism. Replication and intervention research are required before causal or broadly generalizable conclusions can be drawn.

## Data Availability

The raw data supporting the conclusions of this article will be made available by the authors, without undue reservation.
