# Supplementary material for: The pathway to internship success: how academic self-concept and engagement shape outcomes in higher education
Source: Front Psychol. 2026 Oct 2;17:1924391. doi: 10.3389/fpsyg.2026.1924391 (PMC13672491; doi:10.3389/fpsyg.2026.1924391)
Supplement: Supplementary file 1 [file Table_1.docx]

**Supplementary Material**

Supplementary Table S1 reports the full reconstructed item set, dimensions, administration waves, and source mapping for the measures described in the manuscript. Because the source questionnaire was not supplied with the manuscript file, the author must verify every item against the exact wording administered to participants before submission.

***Table S1. Reconstructed questionnaire items, dimensions, administration waves, and source mapping.***

| **Construct** | **Dimension** | **Item wording** | **Wave and source** |
| --- | --- | --- | --- |
| Academic self-concept | Academic competence | ASC1. I understand the academic knowledge required in my field. | Time 1; adapted from [16]-[19] |
|  |  | ASC2. I perform well in coursework related to my major. |  |
|  | Learning confidence | ASC3. I can master difficult concepts in my discipline. |  |
|  | Knowledge application | ASC4. I can apply knowledge from my courses to unfamiliar problems. |  |
|  | Comparative capability | ASC5. Compared with other students in my major, I consider myself academically capable. |  |
|  | Learning confidence | ASC6. I can explain discipline-specific ideas clearly to other people. |  |
| Internship engagement | Behavioral engagement | IE1. I participate actively in the tasks assigned during my internship. | Time 2; adapted from [7]-[9], [21], [23] |
|  |  | IE2. I persist with internship tasks even when they become difficult. |  |
|  |  | IE3. I seek feedback or clarification when I need it. |  |
|  | Cognitive engagement | IE4. I connect workplace problems with knowledge learned at university. |  |
|  |  | IE5. I reflect on why a workplace task succeeded or failed. |  |
|  |  | IE6. I try different strategies when my first approach does not work. |  |
|  | Emotional engagement | IE7. I feel interested in the work I perform during the internship. |  |
|  |  | IE8. I feel that the internship tasks are meaningful for my development. |  |
|  |  | IE9. I feel connected to the workplace learning process. |  |
| Internship outcomes | Performance | IO1. I completed my internship tasks to an appropriate professional standard. | Time 3; adapted from [11], [12], [14], [20], [27] |
|  |  | IO2. I became more effective in handling responsibilities relevant to my field. |  |
|  | Professional skills | IO3. The internship improved my discipline-specific practical skills. |  |
|  |  | IO4. The internship improved my communication and teamwork skills. |  |
|  | Career readiness | IO5. I feel better prepared to enter employment in my field. |  |
|  |  | IO6. The internship clarified the professional expectations of my intended career. |  |
|  | Workplace adaptation | IO7. I adapted successfully to the norms and routines of the workplace. |  |
|  | Satisfaction | IO8. Overall, I am satisfied with the learning outcomes of my internship. |  |
| Auxiliary supervisor rating | Task performance | SR1. The student completed assigned tasks to the expected standard. | Time 3; auxiliary robustness measure |
|  | Reliability | SR2. The student completed responsibilities reliably and on time. |  |
|  | Knowledge application | SR3. The student applied relevant academic knowledge appropriately. |  |
|  | Learning behavior | SR4. The student sought feedback and learned from it. |  |
|  | Overall performance | SR5. The student performed effectively overall during the internship. |  |

***Note. Student-reported items use a five-point scale from 1 (strongly disagree) to 5 (strongly agree). The supervisor rating uses the same five-point anchors. This reconstructed item set is provided for revision purposes and must be checked against the administered questionnaire.***
